# Multifunctional Fructose 1,6-Bisphosphate Aldolase as a Therapeutic Target

**DOI:** 10.3389/fmolb.2021.719678

**Published:** 2021-08-11

**Authors:** David B. Pirovich, Akram A. Da’dara, Patrick J. Skelly

**Affiliations:** Molecular Helminthology Laboratory, Department of Infectious Disease and Global Health, Cummings School of Veterinary Medicine, Tufts University, North Grafton, MA, United States

**Keywords:** aldolase, vaccine, inhibitor, moonlighting function, glycolysis

## Abstract

Fructose 1,6-bisphosphate aldolase is a ubiquitous cytosolic enzyme that catalyzes the fourth step of glycolysis. Aldolases are classified into three groups: Class-I, Class-IA, and Class-II; all classes share similar structural features but low amino acid identity. Apart from their conserved role in carbohydrate metabolism, aldolases have been reported to perform numerous non-enzymatic functions. Here we review the myriad “moonlighting” functions of this classical enzyme, many of which are centered on its ability to bind to an array of partner proteins that impact cellular scaffolding, signaling, transcription, and motility. In addition to the cytosolic location, aldolase has been found the extracellular surface of several pathogenic bacteria, fungi, protozoans, and metazoans. In the extracellular space, the enzyme has been reported to perform virulence-enhancing moonlighting functions e.g., plasminogen binding, host cell adhesion, and immunomodulation. Aldolase’s importance has made it both a drug target and vaccine candidate. In this review, we note the several inhibitors that have been synthesized with high specificity for the aldolases of pathogens and cancer cells and have been shown to inhibit classical enzyme activity and moonlighting functions. We also review the many trials in which recombinant aldolases have been used as vaccine targets against a wide variety of pathogenic organisms including bacteria, fungi, and metazoan parasites. Most of such trials generated significant protection from challenge infection, correlated with antigen-specific cellular and humoral immune responses. We argue that refinement of aldolase antigen preparations and expansion of immunization trials should be encouraged to promote the advancement of promising, protective aldolase vaccines.

## Introduction

**FIGURE 1:**
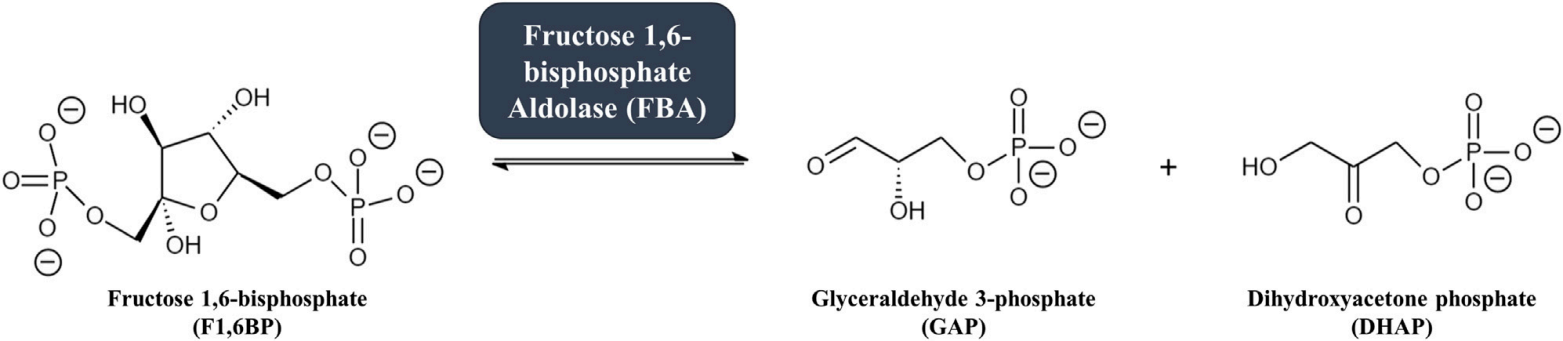
The fourth step in glycolysis: fructose 1,6-bisphosphate aldolase (FBA) catalyzes the reversible conversion of the six-carbon glycolytic enzyme fructose 1,6-bisphosphate (F1,6BP, left) into two three-carbon intermediates glyceraldehyde 3-phosphate (GAP) and dihydroxyacetone phosphate (DHAP, right).

Glycolysis is the highly conserved ten-step metabolic pathway in which glucose is catabolized to pyruvate, leading to the formation of the high-energy molecules adenosine triphosphate (ATP) and reduced nicotinamide adenine dinucleotide (NADH). The fourth step in the glycolytic pathway is the aldol cleavage of the six-carbon fructose 1,6-bisphosphate (F1,6BP) into two triosephosphates: glyceraldehyde 3-phosphate (GAP) and dihydroxyacetone phosphate (DHAP) (Michels and Fothergill-Gilmore, 2017). The reaction, illustrated in Figure 1, is catalyzed by the enzyme fructose 1,6-bisphosphate aldolase (FBA, EC 4.1.2.13), sometimes referred to as simply “aldolase”. The C3-C4 carbon bond in F1,6BP is cleaved to form GAP and DHAP following the formation of an intermediate within the active site of the aldolase enzyme (Cooper et al., 1996; Labbé et al., 2012). Aldolase has been characterized in many bacteria, archaea, and eukaryotes (Stribling and Perham 1973; Yeltman and Harris 1977; Ramsaywak et al., 2004; Galkin et al., 2007) and plays central roles not just in glycolysis but also in gluconeogenesis (the metabolic pathway forming glucose from pyruvate) and fructose metabolism (Droppelmann et al., 2015; Bu et al., 2018; Melkonian et al., 2020).

There are three different classes of aldolases: Class-I, Class-IA, and Class-II. Class-I aldolases utilize a lysine residue within the active site to create a stabilizing Schiff base intermediate with the substrate. Class-I aldolases typically form homotetramers with a total molecular weight of ∼160 kDa (Gamblin et al., 1990; Cooper et al., 1996). In contrast, Class-II aldolases are metal-dependent and rely on divalent ions (typically Zn^2+^) to stabilize the carbanion intermediate formed by the substrate within the active site (Prasad et al., 2013; Michels and Fothergill-Gilmore, 2017). Class-II aldolases typically form homodimers with a total molecular weight of ∼78 kDa but can also form tetramers and octamers (Cooper et al., 1996; Pegan et al., 2009). Class-I aldolases are typically found in higher eukaryotic organisms (e.g. plants and animals), while Class-II aldolases are more common in morphologically less complex eukaryotic organisms (e.g., protozoans, fungi, yeasts, and algae), in prokaryotes and in some archaea (Tunio et al., 2010; Prasad et al., 2013; Falcicchio et al., 2014; Michels and Fothergill-Gilmore, 2017). Some parasitic protozoans, for instance *Trypanosoma brucei* (Dax et al., 2006), *Leishmania donovani* (Gupta et al., 2014), and *Plasmodium falciparum* (Bosch et al., 2007), have been reported to possess Class-I aldolases and not Class-II aldolases. A third class of aldolases, designated Class-IA, is found in archaea (e.g., *Thermoproteus tenax, Pyrococcus furiosus*) (Siebers et al., 2001). These have a similar mechanism of action as Class-I aldolases and have been reported to exist as homo-octamers, decamers or even higher oligomers (Lorentzen et al., 2003; Lorentzen et al., 2004).

Most organisms have an aldolase belonging to one class exclusively, but some bacteria (e.g., *Escherichia coli*, *Mycobacterium tuberculosis*, *Streptococcus pneumoniae*) and algae (e.g., red algae, glaucocystophytes, some diatoms) have been reported to possess both a Class-I and a Class-II aldolase (Tunio et al., 2010; Allen et al., 2012; Shams et al., 2014). The core characteristics of the three classes of aldolase are summarized in Table 1.

**TABLE 1:** A summary of characteristics the three classes of aldolase and the organisms in which they are found.

Aldolase class	Organisms	Characteristics	References
I	Higher eukaryotes (plants, animals), some protozoans (e.g., *Trypanosoma brucei, Leishmania donovani*, *Plasmodium falciparum*), some bacteria, some algae	• Mechanism of action: Schiff base forms substrate intermediate	Gamblin et al. (1990), Cooper et al. (1996), Michels and Fothergill-Gilmore (2017), Prasad et al. (2013), Tunio et al. (2010), Dax et al. (2006), Bosch et al. (2007), Galperin et al. (1998), Allen et al. (2012)
		• Structure: Typically forms homo-tetramers, contains TPI β/α-barrel fold	
IA	Archaea	• Mechanism of action: Schiff base forms substrate intermediate	Lorentzen et al. (2004), Lorentzen et al. (2003), Siebers et al. (2001)
		• Structure: Typically forms homo-octamers/decamers/higher oligomers, contains TPI β/α-barrel fold	
II	“Lower” eukaryotes (some protozoans (e.g., *Giardia lamblia*), fungi, yeasts, algae), most bacteria, some archaea	• Mechanism of action: Divalent ion forms substrate intermediate, metal-dependent	Michels and Fothergill-Gilmore (2017), Prasad et al. (2013), Cooper et al. (1996), Pegan et al. (2009), Tunio et al. (2010), Shams et al. (2014), Galperin et al. (1998), Galkin et al. (2007), Falcicchio et al. (2014), Allen et al. (2012)
		• Structure: Typically forms homo-dimers, contains TPI β/α-barrel fold	

TPI, triosephosphate isomerase

Classical bacterial Class-I aldolases share modest sequence homology with Class-I aldolases of eukaryotes (Thomson et al., 1998). For example, the Class-I aldolase of the bacterium *Staphylococcus carnosus* shares ∼34% amino acid identity with human aldolases, ∼25% with *Drosophila melanogaster* aldolase, and ∼37% with *P. falciparum* aldolase (Witke and Götz 1993). In general, Class-I and Class-II aldolases exhibit no sequence homology (Nakahara et al., 2003) but share similar structural features [a triosephosphate isomerase (TPI) β/α-barrel fold and similar active sites (Galperin et al., 1998) as noted in Table 1], have generally similar sizes (∼40 kDa), and catalyze the same enzymatic reactions. The active sites of Class-I and Class-II aldolases are found in different parts of the TPI barrel, and thus the two classes are considered functionally analogous enzymes that likely evolved independently from one another to perform the same catalytic function using distinct mechanisms of action (Alefounder et al., 1989; Marsh and Lebherz 1992; Nakahara et al., 2003; Shams et al., 2014). Class-IA aldolases show little similarity–as low as 20% at the amino acid level–to classical Class-I or Class-II aldolases (Siebers et al., 2001). However, some important catalytic and substrate-binding residues are conserved between archaean and Class-I aldolases, suggesting that the two protein families share a common evolutionary origin (Lorentzen et al., 2003; Lorentzen et al., 2004). Indeed, some bacterial Class-I aldolases, such as the *E. coli* Class-I aldolase–unusual among the Class-I aldolases–are similar to Class-IA aldolases in sequence, quaternary structure, and biochemical activity (Siebers et al., 2001).

Vertebrates possess three isoforms of Class-I aldolase–aldolase A (ALDOA), aldolase B (ALDOB), and aldolase C (ALDOC), which are encoded by three different genes (Tolan et al., 1987). These isoenzymes are expressed in specific tissues and are similar to one another in molecular mass (∼40 kDa) and catalytic mechanism. ALDOA is expressed mainly in red blood cells and muscle tissue, ALDOB is expressed in the liver, kidney, and small intestine, and ALDOC is expressed in the brain, smooth muscle, and neuronal tissue (Arakaki et al., 2004; Chang et al., 2018). Each isoenzyme has high sequence identity with its counterparts; human ALDOA and ALDOB share 66% identity, ALDOB and ALDOC share 68% identity, and ALDOA and ALDOC share 78% identity (Arakaki et al., 2004). While all three aldolases contribute to the glycolytic pathway, ALDOB and ALDOC have been reported (Chang et al., 2018) to also perform fructolytic functions: i.e., these isoenzymes are additionally able to convert fructose 1-phosphate, derived from the phosphorylation of fructose by fructokinase, into DHAP and glyceraldehyde (Sun and Empie 2012). Class-II aldolase sequence similarities vary wildly: the Class-II aldolase of *E. coli* shares 10% amino acid identity with that of the bacterium *Rhodobacter sphaeroides*, but 49% identity with the Class-II aldolase of the fungus *Schizosaccharomyces pombe* (Mutoh and Hayashi 1994).

**FIGURE 2:**
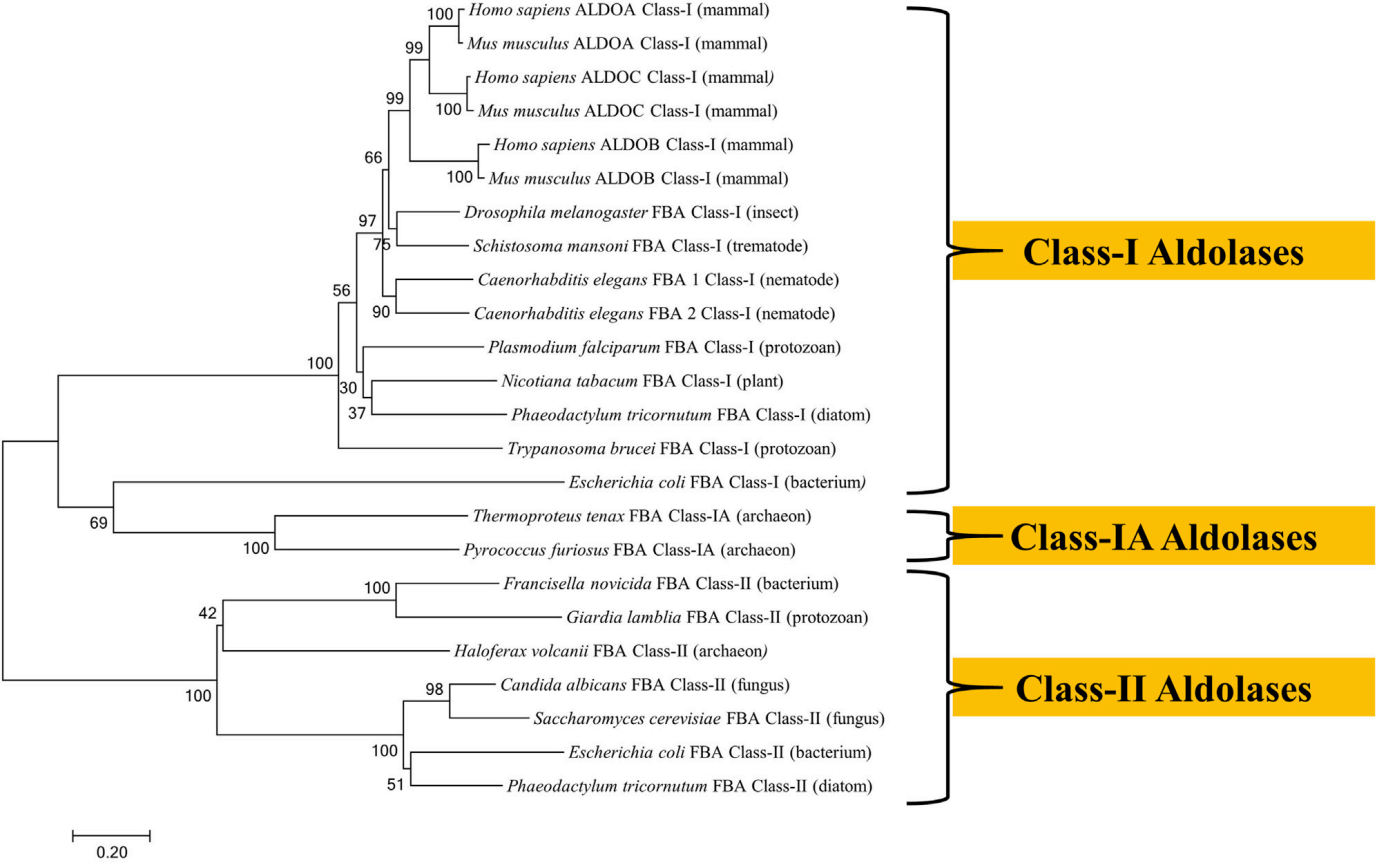
Phylogenetic tree of fructose 1,6-bisphosphate aldolases constructed by Molecular Evolutionary Genetics Analysis (MEGA) Version 7.0.21 software using amino acid sequences obtained from the UniProt database. The sequences were aligned by BioEdit Sequence Alignment Editor software using ClustalW Multiple sequence alignment. The evolutionary history was inferred using the Neighbor-Joining method. The optimal tree with the sum of branch length = 10.38 is shown. The percentage of replicate trees in which the associated taxa clustered together in the bootstrap test (1,000 replicates) are shown next to the branches. The tree is drawn to scale, with branch lengths in the same units as those of the evolutionary distances used to infer the phylogenetic tree. Evolutionary distances were computed using the Poisson correction method and are in the units of the number of amino acid substitutions per site. The analysis involved 24 amino acid sequences. The UniProt accession numbers for the sequences are as follows: *Candida albicans* (Q9URB4); *Caenorhabditis elegans* FBA 1 (P54216); *Caenorhabditis elegans* (P46563); *Drosophila melanogaster* (P07764); *Escherichia coli* Class-I (P0A991); *Escherichia coli* Class-II (P0AB71); *Francisella novicida* (A0A2L1CHL1); *G. lamblia* (O97447); *Haloferax volcanii* (D4GYE0); *Homo sapiens* ALDOA (P04075); *Homo sapiens* ALDOB (P05062); *Homo sapiens* ALDOC (P09972); *Mus musculus* ALDOA (P05064); *Mus musculus*n ALDOB (Q91Y97); *Mus musculus* ALDOC (P05063); *Nicotiana tabacum* (F2VJ75); *Phaeodactylum tricornutum* Class-I (B7GE67); *Phaeodactylum tricornutum* Class-II (B7G4R3); *Plasmodium falciparum* (P14223); *Pyrococcus furiosus* (P58314); *Saccharomyces cerevisiae* (P14540); *Schistosoma mansoni* (P53442); *Thermoproteus tenax* (P58315); *Trypanosoma brucei* (P07752).

The amino acid sequences of aldolase enzymes from a variety of organisms from diverse phyla were aligned using the neighbor-joining method to construct the phylogenetic tree shown in Figure 2. It is clear that Class-I aldolases and Class-II aldolases are phylogenetically distinct entities that cluster into separate branches. Class-IA aldolases are also distinct, but these cluster near the unusual Class-I aldolase of *E. coli* (Siebers et al., 2001).

In this review, we discuss non-traditional functions of aldolases in prokaryotic and eukaryotic organisms. In addition, we review the literature showing that these conserved, classically intracellular proteins can also be commonly found in the extracellular environment and we describe the moonlighting functions of aldolases found extracellularly. Finally, we examine efforts to curtail diseases by inhibiting aldolase using drugs or by targeting the enzymes as vaccines candidates.

## Non-canonical Functions of Intracellular Aldolases

In addition to catalyzing the fourth step in glycolysis, some aldolases can also exhibit other enzymatic properties. For instance, in some archaean species, aldolase acts as a fructose 1,6-bisphosphate phosphatase (Say and Fuchs 2010). Apart from their well-established involvement in carbohydrate metabolism, aldolases have been reported to perform several additional, or “moonlighting”, functions as indeed have other glycolytic enzymes (Jeffery 1999; Shams et al., 2014). Enolase, for example, which catalyzes the eight step in glycolysis, has been reported to possess several non-glycolytic moonlighting functions which could benefit mammalian cells [e.g., stabilizing the mitochondrial membrane (Didiasova et al., 2019)] and pathogens alike [e.g. by binding plasminogen - *Streptococcus pneumoniae* (Bergmann et al., 2001) and *Schistosoma mansoni* (Figueiredo et al., 2015)].

### Intracellular Moonlighting Functions of Aldolase

A key feature of the moonlighting action of aldolase arises from its ability to bind to a wide variety of other proteins. For instance, within some mammalian cells, aldolase can contribute to intracellular scaffolding by e.g., interacting with actin (Wang et al., 1997) and vacuolar-type H^+^-ATPase (V-ATPase) (Merkulova et al., 2011) or by inhibiting Wiskott-Aldrich Syndrome Protein (WASP)-dependent actin polymerization (Lew and Tolan 2013). In the yeast *Saccharomyces cerevisiae*, aldolase helps control RNA polymerase III-directed transcription through a physical interaction (Cieśla et al., 2014). Aldolase has additionally been reported (Rangarajan et al., 2010) to regulate endocytosis by binding to the intracellular trafficking protein sorting nexin 9 (SNX9). Aldolase has also been reported to bind with phospholipase D2 (Kim et al., 2002), heparin (Ta et al., 1999), band three of erythrocytes (Strapazon and Steck 1976), and γ-tubulin (Volker and Knull 1997), but the functional roles of these interactions are unknown.

One remarkable function of aldolase is its ability to serve as a sensor of glucose availability. When the intracellular concentration of the aldolase substrate F1,6BP is low, aldolase has been found to interact with V-ATPase. This promotes the activation of AMP-activated protein kinase (AMPK) and it is this protein that then stimulates ATP production by increasing the activity or expression of proteins involved in catabolism while switching off biosynthetic pathways (Zhang et al., 2017; Li et al., 2019b).

Cancer cells favor aerobic glycolysis as their primary means of energy production in a phenomenon known as the Warburg effect (Vander Heiden et al., 2009). This leads to high expression of many glycolytic enzymes to fulfill the energy demand of synthesizing the cellular metabolites needed for increased replication (Curcio et al., 2021). Overexpression of human ALDOA in pancreatic cancer cells results both in increased rates of glycolysis and increased metastasis, leading to poor prognosis and reduced survival rates (Curcio et al., 2021). In some cancer cells, aldolase can promote DNA-dependent protein kinase (DNA-PK) activation by binding to its catalytic subunit; DNA-PK phosphorylates the tumor suppressor protein p53 to enhance its activity (Ma et al., 2018). Aldolase can also activate the oncogenic Wingless/Int-1 (Wnt) signaling pathway in mammalian cells by blocking the elimination of the signaling molecule β-catenin implicated in promoting colorectal cancer (Caspi et al., 2014). In bladder cancer, ALDOA was reported (Li et al., 2019a) to act as an oncogene by interacting with E-cadherin-epidermal growth factor receptor (EGFR) signaling, leading to cancer cell metastasis. In sum, these studies reveal both pro-oncogenic (*via* Wnt/E-cadherin-EGFR signaling) as well as anti-oncogenic (*via* p53 upregulation) functions for aldolase under different circumstances.

Human ALDOA can bind to the untranslated regions (UTRs) of the Japanese encephalitis virus (JEV) RNA antigenome and can influence viral replication. Expression of the viral nonstructural protein 5 (NS5) reduces to ∼41% in ALDOA-knockout human embryonic kidney (HEK238T) cells (Tien et al., 2014). In addition, JEV infection increased expression of ALDOA by 33% in these cells. ALDOA was similarly found to bind to a stemloop of the 3′-UTR in simian hemorrhagic fever virus, but the function of this binding, if any, is unknown (Maines et al., 2005).

### Moonlighting Functions of Aldolase Inside Pathogens

In the intracellular pathogenic bacterium *Francisella novicida*, aldolase is necessary for bacterial replication within macrophages; the enzyme acts as a transcriptional repressor of the *katG* gene encoding catalase (KatG). When *F. novicida* bacteria escape from the host cell phagosome into the cytosol, bacterial aldolase is upregulated and downregulates *katG* expression. This results in an accumulation of the katG substrate hydrogen peroxide which, in turn, activates the calcium (Ca^*2+*^) transporter TRPM2. A subsequent increase in intracellular calcium activates the macrophage inflammatory response (primarily, production of IL-6) (Shakerley et al., 2016). This outcome is considered beneficial for the bacteria since it creates optimal conditions for bacterial survival by increased dissemination to recruited phagocytes (Ziveri et al., 2017).

In the apicomplexan parasites *P. falciparum*, *T. gondii,* and *Babesia gibsoni*, intracellular aldolase tetramers bind to both intracellular F-actin/myosin filaments (which anchor to the parasite’s inner membrane complex) and to transmembrane adhesins thrombospondin-related anonymous protein (TRAP)/micronemal protein 2 (MIC2) (which bind to host cell surfaces). In this manner, aldolase contributes to parasite motility and host cell invasion (Collingridge et al., 2010; Goo et al., 2013). The TRAP-binding site of aldolase is hypothesized to overlap with its glycolytic active site, and binding leads to inhibition of the enzyme’s classical catalytic function (Buscaglia et al., 2003; Bosch et al., 2007; Boucher et al., 2018). However, the requirement for aldolase in the TRAP/MIC2-binding model for apicomplexan motility and invasion is not seen in *T. gondii*; knockdown and mutation of aldolase did not affect *T. gondii* invasion of human foreskin fibroblast (HFF) cells (Shen and Sibley 2014).

## Evidence for Surface Localization of Aldolase on Many Pathogens

In addition to being widely distributed inside cells, in several instances aldolase has been reported (Gómez-Arreaza et al., 2014; Shams et al., 2014; Pirovich et al., 2019) to be found on a cell’s exterior membranes, including on the host-exposed surface of many pathogenic organisms (both bacteria and eukaryotic parasites). One of the notable mysteries in the study of moonlighting proteins is that many surface-localized proteins, including aldolase, lack transmembrane domains and signal peptides involved in canonical secretion pathways: bioinformatics analysis of 22 published studies on bacterial surface proteomes revealed that >1,000 of 3,619 proteins found on the cell surface lack such domains (Wang and Jeffery 2016). Further study of secretory/surface adhesion mechanisms are needed but there are reported non-classical secretion pathways that may be exploited to export moonlighting glycolytic enzymes; for example, glyceraldehyde 3-phosphate dehydrogenase (GAPDH) has been shown to be secreted by the type III secretion system in *E. coli*, while *S. pneumoniae* enolase associates with the bacterial cell surface using an as-yet-unknown “anchorless” binding mechanism (Wang and Jeffrey 2016).

Extracellular aldolase can interact with host factors and can promote pathogen virulence and survival (Figuera et al., 2013; Oldfield et al., 2017). This surface localization has led many researchers to consider aldolase as a possible drug target or vaccine candidate, given its accessibility. Next, we review the various moonlighting functions of extracellular aldolases that have been reported to aid in the survival of a wide variety of pathogens. We also examine attempts to block aldolase function by targeting these multifunctional enzymes using drugs or vaccines.

**FIGURE 3:**
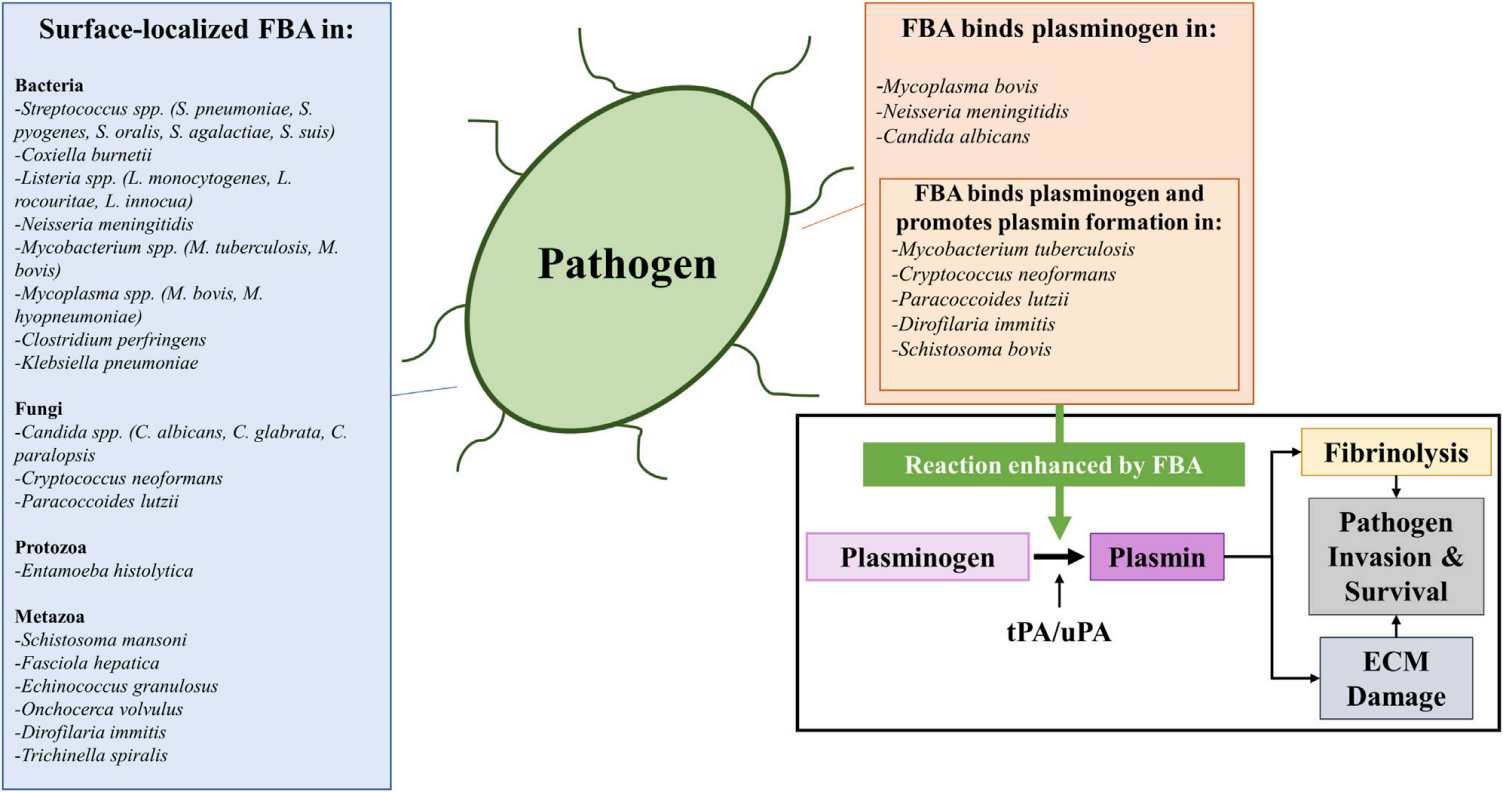
A list of bacterial, fungal, protozoan, and metazoan pathogens reported to possess surface-localized fructose 1,6-bisphosphate aldolase (FBA) (left box) and a list of pathogens whose FBA has been reported to perform a specific moonlighting function: that is of binding plasminogen (top right box) and subsequently promoting its conversion into plasmin (top right box, insert). The conversion of plasminogen into plasmin (illustrated in the bottom right box) is catalyzed by protease activators including tissue plasminogen activator (tPA) or urokinase-type plasminogen activator (uPA). The outcome is fibrinolysis (since plasmin cleaves fibrin blood clots) and/or extracellular matrix (ECM) damage (since plasmin cleaves ECM proteins such as fibronectin and laminin). These mechanisms can be exploited by pathogens to favor their invasion and survival within their hosts *in vivo*, as FBA has been reported to enhance (green arrow) plasmin activation in the presence of activators. Abbreviations: FBA, fructose 1,6-bisphosphate aldolase; ECM, extracellular matrix; tPA, tissue plasminogen activator; uPA, urokinase-type plasminogen activator.

Given the pivotal role aldolase plays in glycolysis–a process that takes place in the cell cytoplasm, it is somewhat surprising to find reports that this highly-conserved enzyme can additionally be found in the extracellular environment. Here, we review the evidence that aldolase can be found in sites other than its traditional location inside the cell. Figure 3 lists those pathogens (which include bacteria, protozoans, fungi, and metazoans) in which aldolase is reported to be surface-localized.

Evidence that aldolase can indeed be extracellular arises from proteomic analysis of isolated membrane preparations of several streptococcal bacteria including *Streptococcus pneumoniae* (Ling et al., 2004), *S. pyogenes* (Severin et al., 2007), *S. oralis* (Oldfield et al., 2017), *S. agalactiae* (Oldfield et al., 2017)*,* and *S. suis* (Wu et al., 2008). Additionally, aldolase has been detected in the secretions of *S. pyogenes* (Lei et al., 2000) and *S. agalactiae* (Oldfield et al., 2017).

Aldolase has been found (Mendonça et al., 2016) at the cell surface of several other bacteria: e.g., *Coxiella burnetii*, the causative agent of Q fever (Flores-Ramirez et al., 2014), and *Listeria* species (including the important foodborne pathogen *Listeria monocytogenes*). Indeed, western blot analysis confirmed (Mendonça et al., 2016) that the hybridoma-derived antibody mAB-3F8, used to detect *Listeria* spp. as well as to distinguish *Listeria* from other bacterial species, binds aldolase in *Listeria* cell wall preparations. In *Neisseria meningitidis* (a causative agent of bacterial meningitis), sub-cellular fractionation and immunoblotting localized (Tunio et al., 2010) aldolase to the outer membrane of the bacterial cell, and this surface accessibility was further confirmed by immunofluorescent localization. Immunofluorescence was also used to confirm (de la Paz Santangelo et al., 2011) a surface-localized aldolase in the medically important bacteria *Mycobacterium tuberculosis*, the causative agent of *tuberculosis*; live bacterial cells could be tagged with an anti-FBA antibody. In addition, these bacteria demonstrated surface aldolase enzymatic activity. *(Mtb. bovis)* was also found to have surface-associated aldolase as revealed by mass spectrometry analysis of a bacterial surface membrane fraction (Pagani et al., 2019). Both *Mycoplasma bovis* and *Mycoplasma hyopneumoniae*, devastating pathogenic agents of bovine bronchopneumonia (*M. bovis*) and porcine enzootic pneumonia (*M. hyopneumoniae*) possess surface-exposed aldolase as revealed by immunofluorescence (Gao et al., 2018), and flow cytometry of live bacterial cells stained with an anti-FBA antibody (Yu et al., 2018), respectively. Immunofluorescence assays confirmed the presence of aldolase on the surface of *Clostridium perfringens*, a causative agent of necrotic enteritis in poultry (Wilde et al., 2019). Cell fractionation and western blot analysis also revealed the presence of aldolase on the surface of the bacterial pathogen *Klebsiella pneumoniae,* a causative agent of disseminated *K. pneumoniae* invasive syndrome–a complication for some diabetics (Lee et al., 2020).

Surface localization of aldolase is not unique to bacterial pathogens: pathogenic fungi in the *Candida* genus (*C. albicans, C. glabrata* and *C. parapsilosis*) express aldolase at the cell surface as determined by mass spectrometry analysis of cell wall surface shavings (Crowe et al., 2003; Chaffin 2008; Karkowska-Kuleta et al., 2019). Other fungal pathogens possessing surface-associated aldolase include *Cryptococcus neoformans* (Stie et al., 2009) and *Paracoccidioides* strain *Pb01* (now classified as *P. lutzii*) (Teixeira et al., 2014; Chaves et al., 2015).

Proteomic analysis of biotinylated surface proteins of parasitic protozoan *Entamoeba histolytica* trophozoites revealed aldolase (along with other glycolytic enzymes–hexokinase, phosphofructokinase, triosephosphate isomerase, glyceraldehyde 3-phosphate dehydrogenase, and enolase) (Biller et al., 2014).

Certain metazoan parasites too have surface-associated aldolase. The aldolase of the parasitic trematode *Schistosoma mansoni* (one of the causative agents of schistosomiasis), for example, was localized *via* immunocytochemistry to both sub-tegumental regions and the tubercles of the schistosome tegument (skin) (El-Dabaa et al., 1998). Tegumental extracts from another parasitic trematode, *Fasciola hepatica,* revealed (Morales and Espino 2012; Ravidà et al., 2016) the presence of aldolase. Immunolocalization assays localized the enzyme to the tegument of adult *Clonorchis sinensis* trematode parasites (Li et al., 2014). Aldolase was identified by western blotting in detergent-dissociated tegumental surface membranes of the protoscolex of the tapeworm *Echinococcus granulosus*, and immunolocalization of fixed *E. granulosus* cysts confirmed the presence of the enzyme in the protoscolex and cyst wall of the parasite (Lorenzatto et al., 2012).

Immune staining using antibodies against recombinant aldolase (rFBA) of the parasitic nematode *Onchocerca volvulus* localized the enzyme to the region of cuticle separation during an L3 to L4 larval molt (McCarthy et al., 2002). Surface-associated antigens collected using a 0.25% cetrimonium bromide-saline solution wash of the filarial parasitic nematode *Dirofilaria immitis* revealed the presence of aldolase among other glycolytic enzymes (glyceraldehyde 3-phosphate dehydrogenase and enolase) (González-Miguel et al., 2013). Finally, immunofluorescent staining of multiple developmental stages of the parasitic nematode *Trichinella spiralis* localized aldolase to the surface of these worms (Yang et al., 2019).

## Moonlighting Functions of Aldolase to Aid Pathogen Survival

Extracellular or surface-associated aldolase has been reported to exhibit a variety of moonlighting functions in pathogenic organisms which have been hypothesized to aid in their invasion, colonization, and/or in immune evasion within their hosts (Gómez-Arreaza et al., 2014). Often, surface-associated or secreted aldolase has been shown to interact with a number of host factors in a manner which could skew host biological pathways (such as the immune response or hemostasis) in a direction that favors the pathogen’s survival.

A commonly reported moonlighting function of the surface-localized aldolase found in pathogens is its ability to interact with plasminogen, a key component of the host coagulation response (Shams et al., 2014; Ayón-Núñez et al., 2018). Plasminogen is the zymogen precursor to the fibrinolytic serine protease plasmin, which degrades fibrin clots. Plasminogen is proteolytically converted to its active form by the proteases tissue plasminogen activator (tPA) or urokinase plasminogen activator (uPA) (Cesarman-Maus and Hajjar 2005). In addition to fibrin clots, plasmin can also degrade components of the host basal membrane and extracellular matrix (ECM), such as fibronectin and laminin (Moser et al., 1993; Bhattacharya et al., 2012). A number of the bacterial species mentioned above have been reported to utilize their surface-localized aldolase to bind plasminogen including *N. meningitidis* (Shams et al., 2016), *M. tuberculosis* (de la Paz Santangelo et al., 2011), and *Mycoplasma bovis* (Gao et al., 2018)—a trait shared by pathogenic fungi (*C. albicans* (Crowe et al., 2003)*, C. neoformans* (Stie et al., 2009), *P. lutzii* (Chaves et al., 2015) and by parasites [*Schistosoma bovis* (Ramajo-Hernández et al., 2007), *D. immitis* (González-Miguel et al., 2013)] alike. Recombinant forms of several of these aldolases were shown to bind to plasminogen by western blotting and/or ELISA (Stie et al., 2009; Chaves et al., 2015; González-Miguel et al., 2015; Shams et al., 2016; Gao et al., 2018), and in several cases, the recombinant aldolases were also shown to enhance the activation of plasminogen into plasmin in the presence of tPA *in vitro* (Stie et al., 2009; de la Paz Santangelo et al., 2011; Chaves et al., 2015; González-Miguel et al., 2015; de la Torre-Escudero et al., 2017). Such binding and activation of plasminogen on pathogen surfaces has been postulated to enhance host tissue damage and facilitate invasion by degradation of the ECM (Bhattacharya et al., 2012), disruption of blood clotting reactions, and to be important for immune evasion [as activated plasmin has additionally been reported to cleave complement components (Ayón-Núñez et al., 2018)] in a process known as “bacterial metastasis” (Lähteenmäki et al., 2005; Degen et al., 2007; Xolalpa et al., 2007; Ayón-Núñez et al., 2018). Figure 2 lists the wide variety of organisms that have been demonstrated to possess an extracellular aldolase (left) and instances in which the enzyme has been shown to bind to plasminogen, promoting its activation (right). Pathogens have been reported to use a number of other glycolytic enzymes, including triosephosphate isomerase (TPI), GAPDH, phosphoglycerate mutase (PGM) and enolase, to bind and activate plasminogen (Pirovich et al., 2019; Pirovich et al., 2020).

Aldolase has also been reported to act as an adhesin in a number of bacterial pathogens, interacting with host receptors to help facilitate invasion. *S. pneumoniae* aldolase binds to the human cell membrane protein flamingo cadherin receptor (FCR); adding recombinant aldolase or anti-FBA antibodies reduced streptococcal adhesion to the human lung cell carcinoma line A549 (Blau et al., 2007). Similarly, recombinant *Clostridium perfringens* aldolase can inhibit adhesion of these bacteria to Caco-2 cells, indicating aldolase’s role as an adhesin in these bacteria too (Wilde et al., 2019). *Mycoplasma bovis* aldolase binds to host cell fibronectin, and this facilitates cell binding to embryonic bovine lung (EBL) cells (Huang et al., 2019). Aldolase-deficient mutants of *N. meningitidis* had a reduced capability to bind to monolayers of human brain microvascular endothelial (HBME) and human larynx carcinoma (HEp-2) cells *in vitro* (Tunio et al., 2010). Blocking surface-localized aldolase of *Klebsiella pneumoniae* with FBA-specific antibodies enhanced bactericidal activity of human neutrophils against the KP-M1 strain of *K. pneumoniae* in an as yet unknown manner (Lee et al., 2020).

Aldolases from pathogens have also been shown to have immunomodulatory effects on their hosts. *Leishmania donovani* aldolase was reported to block IFNγ-induced nitric oxide synthase (iNOS) production by macrophages by specifically binding and activating the macrophage protein Src homology two domain containing protein tyrosine phosphatase-1 (SHP-1) (Nandan et al., 2007). Aldolase found in extract fractions of the parasitic nematode *Trichuris trichiura* was reported to have immunomodulatory effects on peripheral blood mononuclear cells (PBMCs) isolated from naïve individuals *via* stimulation and/or inhibition of the production of Th1 and Th2 cytokines (Santos et al., 2013). These results may present opportunities for novel therapeutics to treat or prevent these infections by blocking the immunomodulatory effects aldolase has on the host.

Table 2 provides a summary of the various functions the aldolase enzyme has been reported to perform (left column) and lists the diverse life forms in which those functions have been reported (middle column).

**TABLE 2:** A summary of enzymatic and non-enzymatic functions that different aldolases have been reported to perform (left column), either in the intracellular (upper section) or extracellular (lower section) environment. The life forms in which those functions have been reported are listed in the middle column.

Location	Functions	Organisms	References
Intracellular	Glycolysis (catalyzes step 4)	Ubiquitous	Michels and Fothergill-Gilmore (2017)
	Gluconeogenesis, fructose metabolism	Ubiquitous	Droppelmann et al. (2015), Lebherz and Rutter (1969), Bu et al. (2018)
	Fructose 1,6 phosphatase activity	Archaea	Say and Fuchs (2010)
	Cellular scaffolding (binds to actin, V-ATPase, WASP)	Mammalian cells	Wang et al. (1997), Lew and Tolan (2013), Chang et al. (2019), Gizak et al. (2019)
	Binds to phospholipase D2	Mammalian cells	Kim et al. (2002)
	Binds to heparin	Mammalian cells	Ta et al. (1999)
	Binds to erythrocyte band 3 protein	Erythrocytes	Strapazon and Steck (1976)
	Binds to g-tubulin	Mammalian cells	Volker and Knull (1997)
	Glucose sensor (binds to v-ATPase)	Mammalian cells	Zhang et al. (2017), Li et al. (2019a)
	Signaling (Wnt, AMPK, E-cadherin-EGFR pathways impacted)	Mammalian cells	Caspi et al. (2014), Li et al. (2019b), Li et al. (2019a)
	Endocytosis control (binds to SXN9)	Mammalian cells	Rangarajan et al. (2010)
	Translational regulation (P53 upregulation, RNA polymerase III-directed transcription, catalase upregulation)	Mammalian cells, yeast, *Francisella*	Ma et al. (2018), Cieśla et al. (2014), Ziveri et al. (2017)
	Viral replication (binds to UTRs)	Japanese encephalitis virus (JEV), simian hemorrhagic fever virus	Tien et al. (2014), Maines et al. (2005)
	Motility complex formation (binds to TRAP in *Plasmodium* spp. + *Babesia gibsoni*/MIC2 in *Toxoplasma* spp.)	Apicomplexans	Collingridge et al. (2010), Nemetski et al. (2015), Goo et al. (2013), Shen and Sibley (2014)
	Blocking iNOS production in macrophages *via* binding to SHP-1	*Leishmania donovani*	Nandan et al. (2007)
	Immunomodulation of PBMCs	*Trichuris trichiura*	Santos et al. (2013)
Extracellular	Plasminogen binding and activation	Bacteria, fungi, metazoan parasites	Ayón-Núñez et al. (2018)
	Host cell adhesion and invasion (binds to FCR, fibronectin)	Bacteria	Tunio et al. (2010), Blau et al. (2007), Wilde et al. (2019), Huang et al. (2019)
	Anti-phagocytic (mechanism unknown)	*Klebsiella pneumoniae*	Lee et al. (2020)
	Downregulates Th1 immunity	*Schistosoma japonicum*	Yan et al. (2020)

Abbreviations: SNX9, sorting nexin 9; V-ATPAse, Vacuolar-type ATPase; WASP, Wiskott-Aldrich Syndrome Protein; AMPK, AMP-activated protein kinase; Wingless/Int-1, Wnt; EGFR, epidermal growth factor receptor; UTRs, untranslated regions; TRAP, thrombospondin-related anonymous protein; MIC2, micronemal protein 2; iNOS, inducible nitric oxide synthase; SHP-1, Src homology 2 domain containing protein tyrosine phosphatase-1; PBMC, peripheral blood mononuclear cells; FCR, flamingo cadherin receptor.

## Aldolase as a Drug Target

The essential nature of aldolase has made it an attractive drug target; abolishing the enzyme’s abilities to 1) drive glycolysis and/or 2) contribute virulence-enhancing moonlighting functions offer an effective solution to staving off disease caused not only by a large and diverse array of pathogens but also by cancers.

### Class-I Aldolase Inhibition

Inhibitors of Class-I aldolases have been used to target cancer cells; the muscle isoform of aldolase (ALDOA) is the most abundant isoform in multiple human cancer types (Chang et al., 2018), and its overexpression predicts poor survival probabilities in patients with lung and pancreatic cancer (Chang et al., 2019; Curcio et al., 2021). A number of ALDOA inhibitors, reviewed by Curcio et al. (2021), had promising anti-oncogenic impact. For example, the inhibitor TDZD-8 1,2,4-thiadiazole was shown to reduce glycolytic activity *in vitro* and *in vivo* in breast cancer cells. While not acting directly on aldolase, the drug inhibited the activity of hypoxia-inducible factor 1-alpha (HIF1α), which mediates the hypoxic response in tumors, and this led to an upregulation in transcription of glycolytic enzymes including ALDOA (Grandjean et al., 2016). The HIF1α inhibitor TX-2098 similarly reduced expression of ALDOA and vascular endothelial growth factor (VEGF) in a xenograft model of pancreatic cancer (Miyake et al., 2012).

Raltegravir, an antiviral compound known to target HIV integrase, can also inhibit the formation of a protein-protein interaction between ALDOA and γ-actin by binding directly to aldolase; the drug has been shown to reduce tumor size and invasive metastasis of lung cancer in mice (Chang et al., 2019). Another compound, “UM0112176”, emulates raltegravir’s mechanism of disrupting ALDOA interaction with actin, disrupting the cytoskeleton and initiating a cascade of intracellular events leading to cancer cell apoptosis (Gizak et al., 2019). A bisphosphonate-based aldolase inhibitor (2-phosphate-naphthalene 6-bisphosphonate) designed to mimic F1,6BP was found to inhibit the proliferation of HeLa cancer cells with an inhibition constant (*K*_i_) of ∼38 nM, while HEK293 cells exhibiting a normal phenotype were not affected (Heron et al., 2018).

The Class-I aldolases of several protozoan parasites have also been considered drug targets (Döbeli et al., 1990; Bakker et al., 2000; Verlinde et al., 2001; Dax et al., 2006). In the case of *Plasmodium berghei*, screening of a library of compounds revealed a candidate (Compound 24) that targeted the aldolase-TRAP complex within the glideosome (a macromolecular complex that helps the parasite move) and inhibited both movement of *P. berghei* sporozoites as well as hepatocyte invasion. Compound 24 inhibited *Plasmodium* aldolase catalytic activity but only when TRAP was present. It is unknown whether Compound 24 binds to aldolase or TRAP, or whether it only binds when the two form a complex (Nemetski et al., 2015). Another candidate (Compound 9, designed to mimic the formation of a Schiff base within the enzyme’s active site) was inhibitory against a range of protozoan parasites with Class-I aldolases; 100 μM Compound 9 blocked recombinant *T. brucei* aldolase enzymatic action (by >99%), recombinant *Leishmania mexicana* aldolase (by 85%), and recombinant *P. falciparum* aldolase (by 70%) but had no appreciable impact on human liver aldolase catalytic function (Dax et al., 2006). The detrimental effects of targeting *T. brucei* aldolase was validated in RNAi knockdown experiments; partial depletion of aldolase was sufficient to rapidly kill trypanosomes *in vitro* by disrupting aldolase activity as well as those of downstream glycolytic enzymes (Cáceres et al., 2009).

### Class-II Aldolase Inhibition

Class-II aldolases belonging to pathogenic microbes have also been considered as pharmacological therapeutic targets due to the structural and mechanical differences they possess compared with the Class-I aldolases of their vertebrate hosts (as reviewed in Table 1) (Capodagli et al., 2014; Semenyuta et al., 2019). The essential nature of aldolase to the viability of these pathogenic organisms has been confirmed by an inability to generate viable mutants with disrupted aldolase gene function *via* mutagenesis. For example, *M. tuberculosis* aldolase knockout bacteria could only be generated in the presence of a rescue copy of the gene (de la Paz Santangelo et al., 2011; Labbé et al., 2012). The Class-II aldolase inhibitory compound phosphoglycolohydroxamate (PGH) (Collins 1974) has been commonly utilized to study both the inhibition and the x-ray crystal structures of Class-II aldolases of a variety of microbial pathogens (e.g., *E. coli* (Hall et al., 1999), *M. tuberculosis* (Pegan et al., 2013)*, Giardia lamblia* (Galkin et al., 2007, Galkin et al., 2009)). However, despite its selectivity for Class-II aldolases, PGH hydrolyzes spontaneously into a toxic byproduct (hydroxylamine) that inhibits other mammalian enzymes (such as triosephosphate isomerase (Collins 1974)), making it, on balance, a poor candidate inhibitor of microbial aldolases for use *in vivo* (Daher et al., 2010).

As Class-II aldolases rely on divalent ions (often zinc or cobalt) as cofactors to stabilize the intermediate formed within the enzyme’s active site, compounds containing metal-chelating groups have been synthesized and tested for their inhibitory nature against aldolases belonging to a variety of pathogenic microorganisms (Daher et al., 2010; Labbé et al., 2012). In one study (Daher et al., 2010), a compound (Compound 1) was designed to both 1) mimic sedoheptulose 1,7-bisphosphate, a substrate of aldolase with a stronger binding affinity to Class-II aldolases than fructose 1,6-bisphosphate, and 2) possess a hydroxamic acid to act as a chelating agent for the zinc ion present in the active site with an electronic density similar to that of the transition state of fructose 1,6-bisphosphate. Compound 1 showed half maximal inhibitory constant (IC_50_) values against aldolases from *M. tuberculosis* and *C. albicans* in the nanomolar range and had high selectivity for Class-II aldolases.

A series of compounds derived from the metal-chelating dipicolinic acid (DPA) were synthesized and tested (Labbé et al., 2012) for inhibition of aldolases belonging to *M. tuberculosis*, *Magnaporthe grisea* (the fungal causative agent of Rice Blast), *Pseudomonas aeruginosa* [an opportunistic bacterial pathogen of immunocompromised patients (Bassetti et al., 2018)], and *Bacillus cereus* [a food-borne bacterial pathogen (Bottone 2010)]. These compounds’ actions were also compared to those of 2,3-dimercaptopropanesulfonate (DMPS)—a chelating agent previously used therapeutically in both animals and humans (Siemann et al., 2003; Flora and Pachauri 2010). The IC_50_ against each aldolase was determined for all inhibitors, with 6-(phosphonomethyl)picolinic acid showing the lowest IC_50_ values: strongest inhibition was against *M. tuberculosis* aldolase, with an IC_50_ of ∼57 μM, while IC_50_ values against *M. grisea*, *B. cereus*, and *P. aeruginosa* aldolases were 78, 78, and 130 μM, respectively. The compound showed no inhibition of rabbit muscle aldolase, confirming its specificity towards Class-II aldolase.

## Aldolase as a Vaccine Target

Sera from individuals infected by pathogens including (but not limited to) *Streptococcus agalatiae* (Fluegge et al., 2004), *Francisella tularensis* (Fulton et al., 2011)*, Campylobacter concisus* (Kovach et al., 2011), *Giardia lamblia* (Palm et al., 2003), *Onchocerca volvulus* (McCarthy et al., 2002), *Schistosoma mansoni* (Harrop et al., 1999), *Schistosoma haematobium* (Mutapi et al., 2011)*,* and *Schistosoma mekongi* (Thiangtrongjit et al., 2018) have been shown to harbor aldolase-specific antibodies. In addition, as detailed below, aldolase has been tested as a vaccine candidate against several of these, and other, pathogens.

### Bacteria

Aldolase has been tested as a vaccine against *Streptococcus pneumoniae*, one of the leading causes of bacterial pneumonia and meningitis worldwide (Portnoi et al., 2006). A cohort of mice immunized (Ling et al., 2004) with recombinant *S. pneumoniae* aldolase and alum adjuvant were subsequently protected against a respiratory challenge with two genetically unrelated strains of *S. pneumoniae* (WU2 and 9VR); 36% of immunized mice survived while 100% of unimmunized control mice died. In other work (Elhaik Goldman et al., 2016), immunization of mice with rFBA in Freund’s adjuvant had a greater protective effect (36% survival rate) than either rFBA + alum (26% survival rate) or a plasmid DNA vaccine containing the aldolase coding DNA (19% survival rate). CD4^+^ T-cells from mice immunized with rFBA + Freund’s that were co-cultured with rFBA-pulsed antigen-presenting cells (APCs) showed significantly increased proliferation compared to similarly treated CD4^+^ T cells from adjuvant-immunized mice. Analysis of cytokines secreted from memory CD4^+^ T-cells from these mice revealed an increase in Th1-type cytokines [e.g., interferon-gamma (IFNγ), interleukin (IL)-12, IL-2, tumor necrosis factor alpha (TNFα)], Th2-type cytokines (e.g., IL-4, IL-5, IL-10) and Th17-type cytokine IL-17A, revealing a robust, mixed immune response to this immunogen. The observed protection may have resulted from the inhibition of moonlighting aldolase function: capsular shedding exposes the bacterial cell wall proteins to host antibodies, which may bind to surface-localized aldolase and neutralize host adhesion (Blau et al., 2007; Elhaik Goldman et al., 2016).

Aldolase has also been tested as a potential vaccine candidate in two fish species important for biomedical research and aquaculture: the zebrafish (*Danio rerio*) and the turbot (*Scopthalamus maximus*), respectively (Sun et al., 2015). In the zebrafish work, recombinant aldolases (emulsified in Montanide™ ISA 763A adjuvant) from a panel of five aquatic, pathogenic bacteria (*Edwardsiella tarda, Aeromonas hydrophila, Vibrio anguillarum, V. harveyi, V. alginolyticus*) isolated from fish were used to vaccinate cohorts of zebrafish. One cohort each was subsequently challenged with one of the fish pathogens. In general, there was a high level of protection in the immunized fish, with relative percent survival (RPS) rates of 50–80% compared to 27% for controls (Sun et al., 2015). Turbot were administered *E. tarda*-rFBA (or PBS as a control) and subsequently challenged with *E. tarda* 4 weeks post-vaccination; vaccinated turbot reached a RPS of 68%, and consistent with zebrafish vaccination data, turbot vaccinated with *E.* tarda-rFBA showed a high antibody level against the aldolases of four other aquatic bacteria, presumably reflecting the high sequence conservation between the bacterial aldolases (75–98%) (Sun et al., 2015). Furthermore, serum from *E. tarda*-rFBA-vaccinated turbot was shown to kill all five bacterial species *in vitro* with ranges of 18.6–52.4% bactericidal capacity. This result, along with the localization of aldolase in the outer membrane led to the conclusion that antibodies raised against rFBA by vaccination bound to aldolase on the surface of these bacteria and enhanced complement-mediated bactericidal activity of the sera (Sun et al., 2015). Increased expression of TLR2, TLR4, TLR5, and MyD88 was seen in whole lysates of the spleen and kidney of vaccinated fish. This is notable as TLRs have been suggested to enhance the adaptive immune response, and the usage of TLR ligands as adjuvants is promising in optimizing the immunogenicity of vaccines (van Duin et al., 2006). Additionally, expression of MHC II and CD4 genes were significantly upregulated compared to MHC I and CD8 genes. Finally, vaccinated fish exhibited increased mRNA expression of IgM in the spleen and kidney, and expression of IL-1β, IL-8, and IL-10 were all upregulated during the early stages post-vaccination. When compared to a previous vaccination trial with the glycolytic enzyme GAPDH, aldolase showed lower antibody titers but a slightly higher RPS, suggesting that the two enzymes may elicit different immune responses and might be combined to increase vaccine efficacy (Sun et al., 2015).

Aldolase vaccines have also been tested against *Clostridium perfringens* in broiler chickens; these immunization experiments used a variety of mechanisms to deliver the antigens to the chickens and examined both the immunoprotective capability of aldolase vaccination and the immune responses of vaccinated birds. Following identification of aldolase as an immunogenic secreted protein unique to virulent isolates of *C. perfringens*, Kulkarni et al. (2007) vaccinated chickens intramuscularly with recombinant *C. perfringens* aldolase before introducing a mild, moderate, or severe oral challenge infection of *C. perfringens*. Vaccination provided significant protection against a mild challenge but not against a moderate or severe challenge (as measured by a reduction in gross intestinal lesion score). However, in a second “mild-moderate” challenge vaccination experiment, it was shown that recombinant aldolase was able to offer significant protection (lower lesion scores). Immunized birds showed high antigen-specific antibody titers, and intestinal antibody responses revealed higher titers of IgY than IgA–likely due to the systemic route of inoculation of the vaccine (Kulkarni et al., 2007).

In other vaccine work (Kulkarni et al., 2008), chickens were immunized orally with an attenuated *Salmonella* vaccine vector expressing either *C. perfringens* aldolase or pyruvate:ferredoxin-oxidoreductase (PFOR) or hypothetical protein (HP) (Kulkarni et al., 2007). After two immunizations over 2 weeks, the birds were challenged with *C. perfringens* through their feed. Protection was once again assessed by gross intestinal lesion scoring, and by analysis of antibodies in sera and intestinal washings of the chickens. Vaccinated birds were significantly protected compared to controls, showing a lower mean lesion score (1.11 on a 0–5 scale), a significant increase in their body weight (mean weight gain of 1.73 kg), and showed a significant increase in antigen-specific antibody titers in their serum compared to pre-immunization levels.

The attenuated *Salmonella* vaccine vector model was employed again (Wilde et al., 2019), here using a strain of *Salmonella* designed to lyse after several rounds of replication and release any expressed *C. perfringens* antigens. Lesion scores were significantly reduced (average lesion score of 0.7) in birds immunized with the FBA-only-producing *Salmonella* strain compared to empty plasmid (average lesion score of 3.1, verifying once more the protective capabilities of aldolase as a vaccine against bacterial infection.

### Protozoans, Fungi, and Metazoan Parasites

Protozoans in the genus *Plasmodium* are the causative agents of malaria, one of the most burdensome parasitic diseases on the planet (Chu et al., 2019). A 41 kDa protein (p41) was identified in as a *Plasmodium falciparum* merozoite surface antigen (Perrin et al., 1985). Immunofluorescence testing detected this protein at the apical end of the merozoite, thought to be located at the rhoptries. Sequence analysis revealed p41 to be aldolase (Certa et al., 1988). The enzyme was localized to the parasite’s cytoplasm in an active form, and to the membrane fractions in an enzymatically inactive form (Knapp et al., 1990). Aldolase (p41) immunization led to reduced parasitemia following *P. falciparum* challenge in monkeys. In this work (Perrin et al., 1985), *Saimiri* monkeys were immunized subcutaneously with purified native p41 in 40 μg of either sodium dodecyl sulfate (SDS) or Nonidet P-40 (NP-40) before being challenged with live parasites *via* tail vein injection. The p41/NP-40-immunized monkeys developed lower peak parasitemia (between 0.008–1.2%) than monkeys immunized with p41-SDS (≤15%). These peaks were lower than those of control monkeys, which were treated with quinine when their parasitemia reached 20%. Since aldolase in NP-40 preparation is more likely to retain its native conformation, this result suggests that this is an important consideration for inducing better immunity against infection. Sera of all p41-immunized monkeys inhibited aldolase enzymatic activity measured *in vitro* by ∼80% (Srivastava et al., 1990). Human adults who have had repeated exposure to *Plasmodium* infection have anti-FBA antibodies in their sera, and this correlates with partial protection against malaria (Perrin et al., 1985; Srivastava et al., 1990).

Aldolase has also been tested as a vaccine candidate against visceral leishmaniasis–a potentially fatal sandfly-transmitted protozoan infection affecting 350 million people in 88 countries (Kaye and Scott 2011). *Leishmania donovani*-challenged hamsters vaccinated with rFBA along with Bacillus Calmette–Guérin (BCG) vaccine showed a significant (∼65%) reduction in parasite load in the spleen, liver, and bone marrow, and gained substantial weight during their trial period when compared to control hamsters immunized with BCG alone or non-immunized hamsters (Gupta et al., 2014). rFBA + BCG-immunized hamsters showed a greater delayed type hypersensitivity response to *L. donovani* challenge than control groups, and macrophages co-cultured with supernatants of lymphocytes from rFBA vaccinated hamsters showed increased production of nitric oxide (NO). Increased expression of Th1 cytokines (IFNγ, TNFα) and iNOS was observed in rFBA-vaccinated hamsters, while Th2 cytokine (IL-4, IL-10) expression was diminished. The rFBA-vaccinated hamsters also exhibited higher IgG2 antibody titers compared to infected control groups, while IgG and IgG1 titers were decreased when compared to infected controls. rFBA-vaccinated hamsters survived longer than their non-immunized or BCG-only immunized counterparts: they remained healthy until their euthanasia 5–8 months post-infection, while the control hamsters succumbed to parasite challenge within two to 3 months after “progressive increase in parasite load.” Lymphocytes of *Leishmania*-cured hamsters (30 days post-treatment with miltefosine) showed a significantly increased lymphoproliferative response compared to lymphocytes from control and infected hamsters when stimulated with rFBA in a lymphocyte proliferation test (LPT). PBMCs of infected or amphotericin B-cured *Leishmania-*infected human patients showed a strong response to rFBA stimulation in LPT assays, suggesting that previous exposure to *L. donovani* aldolase can trigger an immune memory response to the enzyme. PBMCs from endemic contacts (family of infected or cured patients) and cured patients themselves released the Th1-type cytokines IL-12p40 and IFNγ when stimulated with rFBA (Gupta et al., 2014).

Fungi in the genus *Candida* (such as *C. albicans* and *C. glabrata*) are normally commensals in human microflora but become opportunistic pathogens when a human host becomes immunocompromised, leading to invasive candidiasis–one of the leading causes of mortality in both immunocompromised and hospitalized patients (Medrano-Díaz et al., 2018; Mixão and Gabaldón 2020). Aldolase was selected as one of six candidates among *C. albicans* cell wall proteins, to be synthesized into a vaccine. A 14-amino-acid peptide derived from the N-terminus of *C. albicans* aldolase was conjugated onto a β-mannitriose moiety to create the glycopeptide vaccine (β-(Man)_3_-FBA) (Xin et al., 2008). Mice vaccinated with β-(Man)_3_-FBA-pulsed dendritic cells (DCs) generated high FBA-specific antibody titers. Following a lethal challenge of *C. albicans*, immunized mice showed 80–100% survival rates compared to controls. The surviving mice had a significantly reduced fungal colony forming unit (CFU) count in kidney preparations compared to controls. Sera from immunized mice was transferred to naïve mice, which were challenged with *C. albicans*; these mice too showed significantly prolonged survival and reduced CFU counts compared to controls that received non-immunized serum or antiserum preabsorbed with fungal cells. All of the mice that received the FBA peptide-pulsed DCs survived, while <25% of mice immunized with β-(Man)_3_-pulsed DCs survived (Xin et al., 2008).

In later work (Cutler et al., 2011), immunocompromised *Candida*-colonized C57BL/6 mice were intraperitoneally administered a priming multiple antigenic peptide (MAP) aldolase vaccine and a first boost in the form of MAP-pulsed dendritic cells, followed by a MAP/Freund’s adjuvant second boost. All vaccinated mice generated high anti-MAP antibody titers. While in this work protection data were not reported, later experiments (Xin and Cutler, 2011) reported a protective impact of synthetic aldolase MAP injection into mice at the same time as administration of MAP-pulsed dendritic cells. All vaccinated mice exhibited signs of a protective immune response including generation of antigen-specific antibody, prolonged survival, and reduction of viable fungal CFUs in kidneys preparations versus controls (Xin and Cutler 2011). In addition, a MAP/glycan conjugate was used to pulse dendritic cells, and these were administered as a vaccine. This approach also provided protection against candidiasis as characterized by improved survival and reduced CFUs in kidney and brain preparations of infected mice compared to controls. Mirroring the previous study, passive transfer of immune sera from mice immunized with aldolase-pulsed DCs conferred protection on naïve mice. Furthermore, administration of a monoclonal antibody to the aldolase-MAP (called E2-9) also protected mice against candidiasis, demonstrating the importance of eliciting specific antibody responses against cell wall proteins like aldolase in controlling candidiasis. MAb E2-9 was also able to bind to the surface of *Candida* fungal cells as shown by immunofluorescence analysis (Xin and Cutler 2011).

In later work (Xin et al., 2019), the MAP was conjugated to synthetic mimotope peptides used to replace the β-(Man)_3_ glycan moiety. This was undertaken to eliminate the need for oligosaccharide synthesis, thus simplifying vaccine production. Mice that were challenged with *C. albicans* after vaccination with dendritic cells pulsed with these MAP constructs were protected against infection; these MAP-immunized mice showed comparable protection to that exhibited by mice immunized with the original MAP/glycan construct (i.e., they had significantly improved survival and reduced *C. albicans* CFUs in kidney preparations). Again, passive transfer of immune sera generated in this work protected mice from *Candida* infection. Further, IgM and IgG antibodies from vaccinated mice bound to the surface of living fungal cells (Xin et al., 2019).

In other vaccine work (Medrano-Díaz et al., 2018), BALB/c mice were immunized with recombinant *C. glabrata* aldolase in Freund’s adjuvant. Next, mice were inoculated with either *C. albicans* or *C. glabrata via* tail vein injection. Seven days after challenge fungal infection, spleen or liver homogenates generated from the immunized mice were found to yield significantly reduced *C. albicans* and *C. glabrata* CFUs compared to equivalent tissue homogenates from control mice. Anti-*Candida* FBA IgG antibodies from infected human patients have also been tested (Li et al., 2013) for candidemia diagnostic purposes in the laboratory setting with high sensitivity (87%), specificity (93%), as well as positive (77%) and negative (96%) predictive values.

Aldolase has also been used in vaccination experiments targeting multicellular parasites such as the intravascular helminth *Schistosoma mansoni*, a causative agent of human schistosomiasis, affecting 250 million individuals worldwide (Colley et al., 2014; McManus et al., 2018). An immunogenic *S. mansoni* protein (originally designated P44) was found to induce granuloma formation with PBMCs recovered from chronic schistosomiasis patients (Hirsch and Goes 1996; Marques et al., 2008). The protein was subsequently determined to be aldolase and was expressed in *E. coli* as a glutathione-*S*-transferase (GST) fusion protein. This protein (with alum and *Propionibacterium acnes* as adjuvant) was used to immunize C57BL/6J mice (Marques et al., 2008). Two weeks after two vaccine boosts, immunized mice were challenged with 25 *S. mansoni* cercariae (infectious larval-stage parasites); mice were euthanized 8 weeks after parasite infection. Immunized mice generated significantly higher titers of anti-aldolase IgG1 compared to controls, while there was no significant production of anti-aldolase IgG2a in immunized groups. Splenocytes of aldolase-immunized mice produced significantly higher levels of Th2 cytokines IL-4 and IL-10 when compared to splenocytes from control mice when stimulated with aldolase, while levels of the Th1 cytokine IFNγ remained at consistent low concentrations among all groups. There was a significant ∼57% reduction in worm burden in immunized mice, and a significant reduction in the size of hepatic granulomas in the livers of these mice compared to controls (Marques et al., 2008). A 2020 vaccination study demonstrated that immunization with recombinant schistosome GAPDH resulted in increased Th2 cytokines (IL4 and IL-5) production and significant reduction in worm and egg burdens (Tang et al., 2020). This protection was further enhanced by the addition of an anti-CD24 monoclonal antibody, which increased the Th1 response (Tang et al., 2020). This led to the conclusion that a mixed Th1/Th2 response may show synergistic effects in protection against schistosomiasis–could similar protection be elicited by exploiting this synergy when designing other anti-parasite aldolase vaccines?

A DNA plasmid vaccine encoding *S. mansoni* aldolase was injected into CD1 Swiss mice intramuscularly, subcutaneously, or intraperitoneally four times at biweekly intervals (Saber et al., 2013). Mice were infected with 80 cercariae 2 weeks after the last immunization. High titers of anti-aldolase IgG were detected in all groups vaccinated with a right-oriented plasmid relative to those immunized with a control (reverse orientation) plasmid. Reductions in worm burden were as high as 46% in intraperitoneally immunized mice, and these mice also showed reductions in parasite egg burden up to ∼40% in both the liver and intestine. Mice injected subcutaneously had worm burden reductions of 29% while intramuscular injection of the vaccine reduced worm burden by only 15%, which was not statistically significantly different from the control situation. Egg burdens in subcutaneous and intramuscular vaccination groups were not significantly reduced compared to controls. In all cases, the mean granuloma diameter and the granuloma number in aldolase-immunized mice was significantly reduced compared to vector-alone vaccinated controls, and granulomas of aldolase-immunized mice were characterized by high levels of fibrosis, increased numbers of lymphocytes, and dead miracidia (i.e., larvae within the eggs) (Saber et al., 2013). In comparison, DNA plasmid vaccines coding for schistosome triosephosphate isomerase (TPI), another surface-localized glycolytic enzyme, have been used in a variety of animal models. For example, one study demonstrated reduced worm and egg burdens and an increased Th1-polarized response (characterized by increased levels of IL-2, IFNγ, and TNFα) in mice immunized *via* electroporation with a codon-optimized DNA vaccine (Zhu et al., 2010). It is possible codon optimization and the differing route of administration led to the distinct immune response, and further studies on aldolase vaccines using these tools may offer new insights into protective vaccine development.

*Onchocerca volvulus* is a parasitic filarial nematode that causes the debilitating ocular disease onchocerciasis (also known as river blindness) that affects ∼18 million people globally (Etya’ale 2001). BALB/cByJ mice subcutaneously injected with urea-solubilized recombinant *O. volvulus* aldolase in Freund’s were surgically implanted with a subcutaneous diffusion chamber containing *O. volvulus* L3-stage larvae (McCarthy et al., 2002). There was a ∼50% reduction in larval survival 21 days later in the aldolase-immunized mice compared to controls. While antibodies from sera of nonendemic control human subjects (n = 22) did not recognize recombinant aldolase by western blot analysis, the sera of 88% of *O. volvulus*-infected subjects (n = 42) and 68% of putatively immune individuals (n = 34, based on previous clinical history and exposure to infection) recognized the enzyme. Recombinant aldolase was able to induce T-cell proliferation, and production of IL-5 and IFNγ in PBMCs collected from putatively immune and infected individuals but not from non-endemic, control individuals. Since the protection seen in the mouse model may not predict human vaccinations outcomes, using larger animals such as non-human primates as vaccine models may replicate human immune responses *in vivo* more closely (McCarthy et al., 2002). Partial protection from *O. volvulus* was also demonstrated in mice immunized with a DNA vaccine coding for GAPDH, which stimulated a pre-challenge Th1 response and induced increased generation of GAPDH-specific IgG and IgM antibodies (Steisslinger et al., 2015).

*Trichinella spiralis* is a parasitic nematode that causes trichinellosis when a human consumes uncooked, infected animal muscle tissue containing infective larvae (Gottstein et al., 2009). Trichinellosis occurs globally, with estimates of ∼10,000 cases yearly (Rawla and Sharma 2020). Recombinant *T. spiralis* aldolase in ISA206 adjuvant was injected subcutaneously into BALB/c mice, and the mice were orally challenged with 300 *T. spiralis* muscle larvae 2 weeks after the last of two boosts (Yang et al., 2019). These mice showed a significant reduction in adult worm (48.7%) and muscle larval (52.5%) burden compared to controls. Total serum anti-FBA IgG titers were significantly increased in aldolase-immunized mice compared to control mice, and the IgG1 level was higher than that of IgG2a. Specific IgE titers were also increased following aldolase immunization, leading to the suggestion that expulsion of worms from the intestine may be at play, as IgE-dependent mast cell activation has been promoted as a contributing factor to the elimination of gastrointestinal nematode parasites (Mukai et al., 2016). A mixed Th1/Th2 response induced by vaccination was confirmed by cytokine analysis: significantly increased IL-2, IFNγ, IL-4, and IL-10 levels were all detected in the supernatants of aldolase-stimulated splenocytes from immunized mice, once more demonstrating the synergistic effects of the balance between Th1/Th2 immune responses in protecting against infection. Further research is required to discover the precise mechanism by which aldolase elicits a protective response against *T. spiralis* (Yang et al., 2019).

In summary, aldolase as an immunogen has generated protective immunity in a wide variety of vaccination trials targeting bacteria, fungi, protozoans, and metazoan parasites. Table 3 provides a summary of these various immunization efforts and their outcomes. While vaccination against pathogens has generally focused on generating a protective immune response in the host, it may be of interest to researchers conducting future trials to examine the effects vaccination has against moonlighting functions. As previously mentioned, anti-aldolase antibodies have been shown to diminish moonlighting functions [e.g., reducing streptococcal binding to mammalian cells (Blau et al., 2007)], so it is plausible that protection observed in vaccination trials may partially stem from the curbing of vital moonlighting functions. Further research is required to fine-tune immunogen and adjuvant preparations (e.g., by introducing TLR agonists and/or enhancing vaccine design and routes of administration) in order to elicit an optimal protective immune response (e.g., perhaps a mixed Th1/Th2 responses when needed).

**TABLE 3:** A summary of experimental vaccination trials using aldolase as an immunogen against pathogenic bacteria, protozoans, fungi, and metazoan parasites. The pathogenic species, vaccine preparations, and animal models used in each study are listed. The Outcomes column summarizes the main protective and immunological consequences recorded in aldolase-vaccinated animals versus controls.

Group	Species	Vaccine preparation	Animal model	Outcomes	References
Bacteria	*Streptococcus pneumoniae*	Recombinant protein	Mouse	Improved survival (26–36%), robust, mixed Th1, Th2, Th17 (IFNγ, IL-12, IL-2, TNFα, Il-4, IL-5,IL-10, IL-17A) immune response	Ling et al. (2004), Elhaik Goldman et al. (2016)
	Aquatic bacteria (*E. tarda, V. anguillarum, V. harveyi, V. alginolyticus, A. hydrophila*)	Recombinant protein	Fish	Increased survival (50–80%), upregulated immune gene (TLR2, TLR4, TLR5, MYD88) expression, increased specific antibody and cytokine (IL-8, IL-10, IL-1β) production	Sun et al. (2015)
	*Clostridium perfringens*	Recombinant protein, *Salmonella* vector	Chicken	Improved lesion scoring, increased specific IgY, IgM, IgA antibody titers, improved body weight, increased lymphocyte proliferation	Kulkarni et al. (2007), Kulkarni et al. (2008), Wilde et al. (2019)
Protozoa	*Plasmodium falciparum*	Native protein	Monkey	Reduced parasitemia, specific antibody generation	Perrin et al. (1985)
	*Leishmania donovani*	Recombinant protein	Hamster	Reduced parasite load, increased weight gain, prolonged survival, increased Th1 gene expression and cytokine (IFNγ, TNFα) response, downregulated Th2 gene expression, increased specific IgG antibody generation	Gupta et al. (2014)
Fungi	*Candida albicans*	Multiple antigen peptide (MAP)-pulsed dendritic cells, MAP conjugated to synthetic mimotope peptides	Mouse	Specific antibody generation, prolonged survival, reduced tissue fungal load	Xin et al. (2008), Cutler et al. (2011), Xin and Cutler (2011)
	*Candida glabrata*	Recombinant protein	Mouse	Specific antibody generation, reduction of CFUs from tissue homogenates	Xin and Cutler (2011), Medrano-Diaz et al. (2018), Xin et al. (2019)
Metazoa	*Schistosoma mansoni*	Glutathione-S-transferase fusion protein, DNA plasmid	Mouse	Specific IgG antibody generation, Th2 (IL-4, IL-10) cytokine production, worm (15–57%) and egg burden (0–40%) reduction, granuloma size and number reduction	Marques et al. (2008), Saber et al. (2013)
	*Onchocerca volvulus*	Recombinant protein	Mouse	Reduced larval survival (50%), specific antibody generation, T-cell proliferation, enhanced cytokine (IL-5, IFNγ) production	McCarthy et al. (2002)
	*Trichinella spiralis*	Recombinant protein	Mouse	Reduction in worm burden (∼49–53%), specific IgG and IgE antibody generation, mixed Th1/Th2 immune response (IL-2, IFNγ, IL-4, IL-10 generation)	Yang et al. (2019)

Abbreviations: MAP, multiple antigen peptide; Th, T helper; IL, interleukin; Ig, immunoglobulin; TNF, tumor necrosis factor; immunoglobulin; CFU, colony forming unit

### Autoimmune Disease

Aldolase has been described as an autoantigen in patients with some autoimmune disorders including Alzheimer’s disease (Mor et al., 2005), atypical movement disorders (Privitera et al., 2013), and autoimmune retinopathies (Adamus 2017). Antibodies in serum from Alzheimer’s patients, as well as some patients with multiple sclerosis and healthy controls, bind to aldolase A (ALDOA) (Mor et al., 2005). These antibodies also reduce the enzymatic activity of aldolase *in vitro*. To investigate if this anti-aldolase immunity might contribute to Alzheimer’s disease pathology in an animal model, Lewis rats, C57BL mice, and NOD mice were immunized with rabbit muscle aldolase A in Freund’s adjuvant (Mor et al., 2005). However, this resulted in no overt pathology; no animals showed clinical signs of CNS or muscle disease, and histological analysis of organs (brains, muscle, spinal cord, heart) showed no signs of inflammation. These results have led to the conclusion that aldolase autoantibodies represent a nonspecific biomarker of Alzheimer’s disease and would be a poor immunotherapy candidate for this disease (Wu and Ling, 2016).

Interestingly, intraperitoneal administration (Yan et al., 2020) of recombinant *Schistosoma japonicum* aldolase in female non-obese diabetic (NOD) mice ameliorated induced type 1 diabetes severity and incidence (Yan et al., 2020). rFBA-treated animals had significantly lower diabetes incidence (40% reduction) and reduced blood glucose levels compared to control mice. These effects were ascribed to Th2 type response induction following rFBA injection, characterized by increased generation of the anti-inflammatory cytokine IL-4 and downregulation of the pro-inflammatory Th1 cytokine IFNγ in the spleen and pancreatic lymph nodes. Furthermore, rFBA-injected mice exhibited increased Treg cell numbers and an associated increase in IL-10 and TGFβ detected in the spleen and lymph nodes compared to control mice. Immunohistochemical analysis of pancreatic islets revealed that rFBA-treated mice had reduced lymphocyte infiltration and improved insulin secretion. These results led to the conclusion that rFBA injection induced anti-inflammatory Th2 immune polarization and downregulated the pro-inflammatory Th1 response, resulting in attenuated inflammation of pancreatic islets and thus ameliorating type I diabetes pathology.

## Conclusion

Given its key role in the glycolytic pathway, fructose 1,6-bisphophate aldolase has long been known to play a central role in the cell’s internal glucose metabolism. More recently, this conserved protein has been discovered to possess a myriad of other, non-glycolytic functions both inside and outside the cell. In an extracellular location, aldolase has been shown to possess numerous moonlighting functions for pathogens such as binding to host cell receptors to facilitate invasion or activating plasminogen to potentially modulate host hemostasis and enhance survival.

Given its immunogenic nature in pathogen-infected human patients and its surface localization in many pathogens, aldolase has become a focus of drug discovery as well as a candidate in several animal-based vaccine studies. Drugs designed to inhibit aldolase are selected for their ability to disrupt proper function of the active site, whether that be designing a competitor that mimics the Schiff base, or a chelator that removes stabilizing metal cofactors in the cases of Class-I and Class-II aldolases, respectively. As discussed above, several Class-I and Class-II aldolase inhibitors have been identified and further work is required to refine their potency and specificity. Elucidating and targeting the various non-canonical mechanisms and pathways that pathogens use to secrete and traffic moonlighting aldolase to the extracellular environment could also be helpful in the development of new strategies and tools to combat infection (Jeffery 2018).

Aldolase vaccination studies involving bacteria, fungi, or parasites all invariably resulted in the development of varying degrees of protection from challenge infection, and this was associated with induced cellular and/or humoral immune responses. Novel immunoinformatics approaches to predict optimal peptide sequences with strong binding affinities to host MHC molecules while minimizing allergic reactivity may prove useful in simplifying the search for specific antigenic epitopes within the enzyme structure (Mohammed et al., 2018; Elhag et al., 2020). The development of new technologies such as immunoinformatics, the exploration of new vaccine designs and alternative routes of immunogen administration (i.e., protein versus DNA versus RNA, oral administration versus intramuscular etc.), as well as the expansion of aldolase vaccination trials and subjects in general should be undertaken to further advance efforts to generate clinically useful aldolase vaccines in order to reduce pathogenic disease and improve global health.
